# QSAR Models for Active Substances against *Pseudomonas aeruginosa* Using Disk-Diffusion Test Data

**DOI:** 10.3390/molecules26061734

**Published:** 2021-03-19

**Authors:** Cosmin Alexandru Bugeac, Robert Ancuceanu, Mihaela Dinu

**Affiliations:** 1 Faculty of Pharmacy, Carol Davila University of Medicine and Pharmacy, 6 Traian Vuia Street, Sector 2, 020956 Bucharest, Romania; bugeac.alexandru.cosmin@gmail.com; 2 Department of Pharmaceutical Botany and Cell Biology, Faculty of Pharmacy, Carol Davila University of Medicine and Pharmacy, 6 Traian Vuia Street, Sector 2, 020956 Bucharest, Romania; mihaela.dinu@umfcd.ro

**Keywords:** pseudomonas, antimicrobial, QSAR, chemical descriptors, machine-learning, KNN, support vector classifier, AdaBoost

## Abstract

*Pseudomonas aeruginosa* is a Gram-negative bacillus included among the six “ESKAPE” microbial species with an outstanding ability to “escape” currently used antibiotics and developing new antibiotics against it is of the highest priority. Whereas minimum inhibitory concentration (MIC) values against *Pseudomonas aeruginosa* have been used previously for QSAR model development, disk diffusion results (inhibition zones) have not been apparently used for this purpose in the literature and we decided to explore their use in this sense. We developed multiple QSAR methods using several machine learning algorithms (support vector classifier, K nearest neighbors, random forest classifier, decision tree classifier, AdaBoost classifier, logistic regression and naïve Bayes classifier). We used four sets of molecular descriptors and fingerprints and three different methods of data balancing, together with the “native” data set. In total, 32 models were built for each set of descriptors or fingerprint and balancing method, of which 28 were selected and stacked to create meta-models. In terms of balanced accuracy, the best performance was provided by KNN, logistic regression and decision tree classifier, but the ensemble method had slightly superior results in nested cross-validation.

## 1. Introduction

*Pseudomonas aeruginosa* is a Gram-negative bacillus, widespread in various environments, from soil to water and from plants to animals [1]. Whereas in healthy people it seldom triggers disease, in patients with a weakened immune system it may quickly proliferate and trigger a range of serious acute and chronic infections, being an opportunistic pathogen [1,2]. It is the critical pathogen responsible for the morbidity and mortality associated with cystic fibrosis, as well as one of the major microbes causing nosocomial infections [3]. It is one of the six “ESKAPE” (*Enterococcus, Staphylococcus, Klebsiella, Acinetobacter, Pseudomonas, Enterobacter*) microbial species, characterized by their remarkable ability to “escape” the usual antibiotics and it belongs to the World Health Organization “critical” list of bacteria for which developing new antibiotics should be the highest priority [4]. Its adaptability and resilience, favored by an abundance of regulatory genes in a large genome, its high number of virulence factors and its ability to withstand most antibiotics places this bacteria among the dreadful microbial pathogens [5]. Although bloodstream infections by *Escherichia coli* and *Klebsiella* spp. are more frequent than those of *P. aeruginosa*, the latter is associated in a consistent manner with higher mortality (23–36%) [6]. Considering the ability of *P. aeruginosa* to escape currently used antibiotics, there is a strong need of developing new such antibacterial products, active against Gram-negative germs and, particularly, against *P. aeruginosa* [7].

The introduction of antibiotics in therapy has marked unprecedented progress in the control of disease and reduction of mortality in human history, conservative estimates indicating death reductions by 25–75% for different diseases [8]. The development of new antibiotics has remained a challenge in the last decades, with low yields despite impressive progresses in certain areas of drug development [9]. During the 1990s and 2000, the number of new antibiotic drugs approved by the world’s key regulatory agencies has suffered a steep decline [10]. Large pharmaceutical companies tend to abandon their antibiotic research programs and turn their back on developing new antibiotics because of financial risks, modest returns and low probability of development success [11]. 

Drug design and development in the classic approach has been a toilsome, high-priced, time-consuming and complex activity [12]. Progress in the computational field has allowed the drug discovery processes to become more efficient and less costly, with a variety of structure-based or ligand-based approaches used in this respect [13]. Among the ligand-guided approaches, QSAR methods are very popular, in this sense being stated that “one would say that nowadays no drug is developed without previous QSAR analyses” [14]. They are computational methods that attempt creating relationships between chemical structure features of a set of compounds and one of their biological activities expressed numerically [15]. The practical applications and uses of QSAR span a wide range, from establishing structural requirements for the prospective ligands to finding new prospective compounds via virtual screening and to estimation of ADMET (Absorption, Distribution, Metabolism, Excretion, Toxicity) features of a large number of chemical compounds [16]. Valid QSAR models allow virtual screening of large and very large databases of chemical compounds, resulting in identification with meager costs of chemical compounds with a high potential of being active and satisfying the preconditions of promising drugs [17]. Whereas probably the majority of QSAR models tend to focus on a single target, a smaller number of models use a multi-target (or multi-objective) approach. The multi-target models have been appreciated for their many potential advantages, from the prediction of biological activity without being narrowly limited to a single protein to the evaluation of complex pharmacological or toxicological profiles based on many experimental settings [18,19,20].

It has been recognized that in assembling a proper training set, it is necessary to cover a wide chemical diversity; public databases, such as ChEMBL or PubChem, are most useful in achieving a diverse chemical space for the modeling exercise [12]. However, as a group of researchers investigating such public databases has stated, “there are no databases where we have not found errors” [21]. In a number of cases, wide variability is observed in activity values reported for the same chemical compound in different experiments, depending on the source of the included reports [22,23]. One of the methods widely used to assess antimicrobial susceptibility is the disk diffusion, because it has a number of advantages (simple to carry out, good reproducibility, inexpensive and without needing sophisticated equipment, easily understood by clinical practitioners and quite flexible) [24]. In ChEMBL, for *Pseudomonas aeruginosa* as a target, the number of bioactivities based on the disc diffusion method (inhibition zone (IZ) as the “standard type” 7056 data points as of 18 January 2020) is only second to those based on the minimum inhibitory concentration (MIC) and more extensive than other measurements (such as “activity”, “inhibition”, “MIC90” etc.). Whereas MIC values against *Pseudomonas aeruginosa* have been used previously for QSAR model development, we could find no previous attempts of using IZ measurements for this purpose in the literature and decided to explore their use in this sense. Because “disk diffusion susceptibility testing…provides only a qualitative result” [24], we used classification and not regression machine learning methods. Thus, we report on QSAR models developed for substances active against *Pseudomonas aeruginosa*, using IZ values from the ChEMBL database. 

## 2. Results

**Figure 1:**
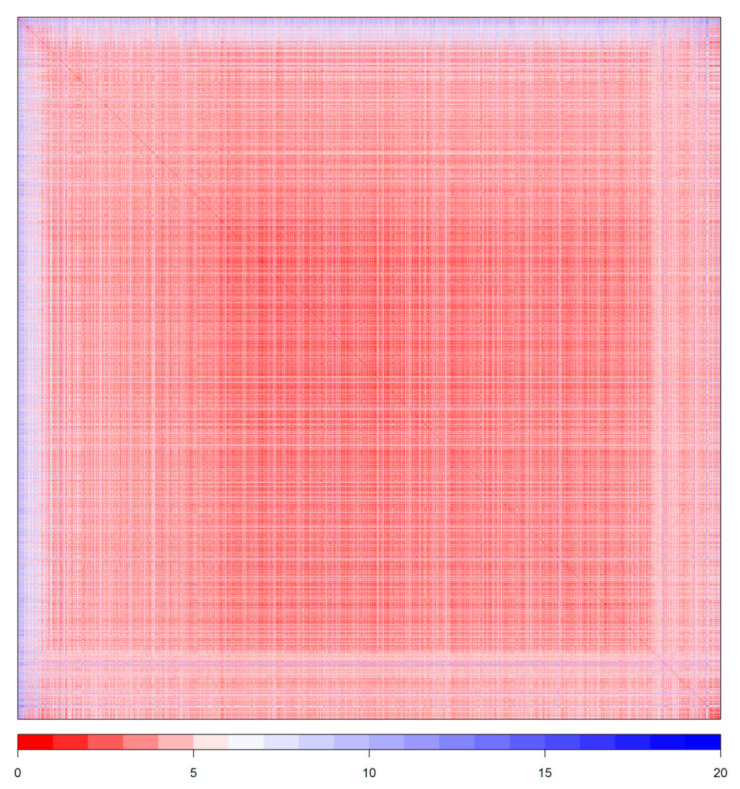
Dissimilarity plot based on the Tanimoto coefficient for the training data set used in this study. Lower values (more intense red) indicate lower dissimilarity (zero values showing identity the diagonal line). Spectral seriation was used between clusters and multidimensional scaling (“MDS”) within clusters.

The training data set consisted of 3226 observations, with IZ values varying between 0 and 54 mm. The data set’s chemical diversity, estimated through the Tanimoto similarity coefficient computed based on the 166-bit MACCS fingerprints, is represented graphically in Figure 1. The median value of each compound’s median similarity to the others (i.e., median of all column/row medians of the symmetric Tanimoto matrix) was 41.67%.

### 2.1. Performance of Models Built with Molecular Descriptors

**Figure 2:**
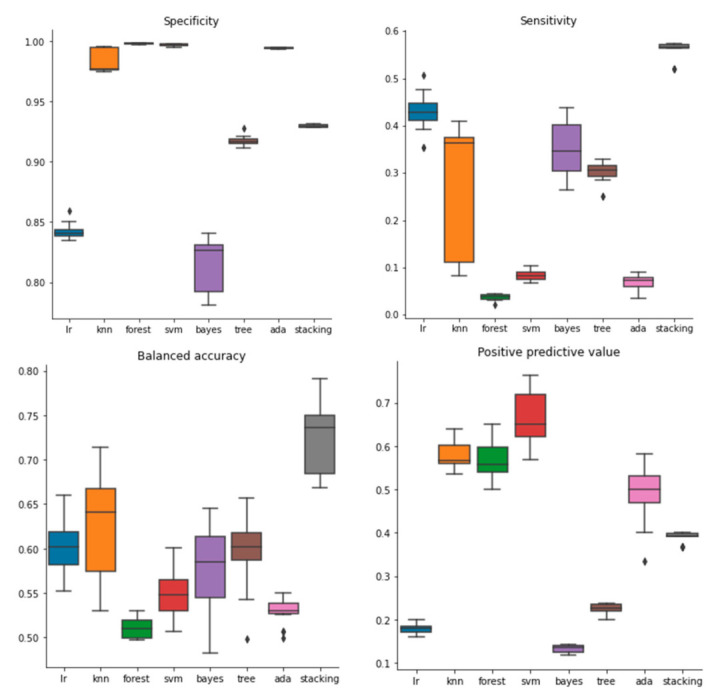
Performance of individual QSAR models and meta-model in the nested-cross validation.

In total, 28 models were created with the molecular descriptors (seven classification algorithms and four feature selection methods with hyperparameter tuning in the inner loop of a nested cross-validation process) and stacked to create a meta-model. The latter was built by applying the logistic regression algorithm to the predicted probabilities of the individual models. Different values for the decision threshold were explored and the best performance was observed for a probability threshold of 0.65. Using a higher value for the threshold led to an increase in positive predictive value with a cost in balanced accuracy and sensitivity. In nested-cross validation, individual models had a balanced accuracy (BA) varying between 48.21% and 79.11%; the stacked model had a mean BA of 72.61% (s.d. 4.61%) (Figure 2, Table S3). The models had good specificity but performed rather poorly in terms of sensitivity, a trade-off we were willing to accept, considering the imbalance between active and inactive compounds in the data set. Specificity varied between 78.09% and 99.83%; the stacked model had a mean of 92.96% (s.d. 0.12%). Sensitivity values ranged between 3.47% and 57.46%, with best values for the stacked model (mean 56.01%, s.d. 2.06%). From our perspective, a very high specificity coupled with a lower sensitivity was to be preferred, given the imbalanced nature of the data set. It leads to a higher positive predictive value (a smaller proportion of the active compounds is predicted correctly, but those predicted have a higher probability of being active). PPV ranged between 11.72% and 76.31%; the stacked model had a mean of 38.99% (s.d. 1.19%). Although other models had higher PPV (e.g., SVM models over 70%), those had a very low sensitivity (a mean sensitivity of 0.4% for one SVM model). Because the dataset consisted of only about 8% compounds classified as “active”, the relatively low PPV of 38.99% achieved with the stacked model implies an improvement of over 400% (compared to what is to be expected by mere random labeling). Other attempts of building meta-models were made with different algorithms (random forests, support vector classifier, k nearest neighbors and decision trees), but the performance of those meta-models (in nested cross-validation) was inferior to the one using logistic regression.

As was to be expected, the performance in nested-cross validation was much better on the data sets balanced through oversampling, undersampling, or SMOTE (28 models for each of these), but because these data sets are artificial, in this case, these results are much less relevant (Tables S6–S8); what really counts is the results on the external test set. Not surprisingly, for most algorithms and features used, the test set results did not differ substantially from those seen with the models where the imbalance was dealt with through class weights or a loss function. However, two of the models developed with the downsampling techniques had a balanced accuracy on the external data set higher than 70%, i.e., higher than that seen with the “native data” models, except for the stacking model; they were built with the knn and svm algorithms (Table S9). None of the individual models developed with the naïve random oversampling technique had a balanced accuracy value higher than 64.54% on the external data set, but the stacked model developed with these models had a balanced accuracy of 76.05% (Table S10). Among the models developed with the help of the SMOTE technique, four models had for the balanced accuracy on the external data set values higher than 73%, three of them higher than the one obtained with the stacking model built with the “native data”, all of them being built with the knn algorithm (Table S11). However, all four of these SMOTE models had PPV values lower than those observed for the stacked model built with the native data (27.11–33.73%, versus 40%).

### 2.2. Performance of Models Built with Molecular Fingerprints

The performance obtained with the MACCS fingerprints was slightly inferior to that obtained with the Mordred descriptors: in the nested cross-validation, the mean balanced accuracy varied between 50.17% (no better than random) for the naïve Bayes algorithm to 66.42% (for the decision tree algorithm) and it increased to 68.16% for the ensemble model (Table S12). The results on the external data set are shown in Table S13. As expected, the nested cross-validation performance increased considerably with the downsampling, oversampling and SMOTE data sets, but for these, the only results really relevant are those on the external data set. A single algorithm based on the downsampling technique reached a balanced accuracy higher than 70% (decision trees algorithm with mutual information-based feature selection) and two additional models got balanced accuracy values higher than 65% (also based on decision trees Tables S14 and S15). With the naïve random oversampling, the highest individual model had 67.66% balanced accuracy and the stacked model 71.27% (Tables S16 and S17). For the SMOTE models, only two had balanced accuracy values higher than 65% and the highest one was 66.64%. These values are not superior to the values obtained with the models built on the native data (70.99% for the ensemble model, 65.31% and 65.33% for two decision tree models) (Tables S18 and S19). 

The performance of the models built with the Morgan fingerprints was also very similar to the one obtained with the MACCS: the balanced accuracy varied between 50.78% (AdaBoost algorithm) and 63.69% (decision tree algorithm) and it increased to 65.93 for the ensemble model (Table S20). The results on the external data set are shown in Table S21. Models built with artificial data sets based on downsampling, oversampling and SMOTE had similar performance to those obtained with MACCS fingerprints on the external data set. In terms of balanced accuracy, with downsampling and naïve random oversampling three models for each technique had values higher than 65% on the external data set (maximum 68.89% Tables S22–S25) and with SMOTE two models had values higher than 65% on the external data set (Tables S26–S27).

Saagar fingerprints were also not associated with a sizeable increase in performance over the Mordred descriptors, or MACCS and Morgan fingerprints. In the models built with the native data set, the highest balanced accuracy (in nested cross-validation) was observed for the stacked model (72.71%, s.d. 0.04), very similar to the one obtained with the native set of Mordred descriptors. The two individual models with the highest balanced accuracy (in nested cross-validation) were based on decision trees (65.74% and 68.50%) (Tables S28–S29). The use of downsampling, oversampling, or SMOTE techniques did not result in considerably improved performance on the external validation set: the highest balanced accuracy values were 69.83% (downsampling, random forest algorithm), 75.63% (oversampling, stacked model) and 71.52% (SMOTE, decision tree) (Tables S30–S35). 

### 2.3. Y-Randomization

**Figure 3:**
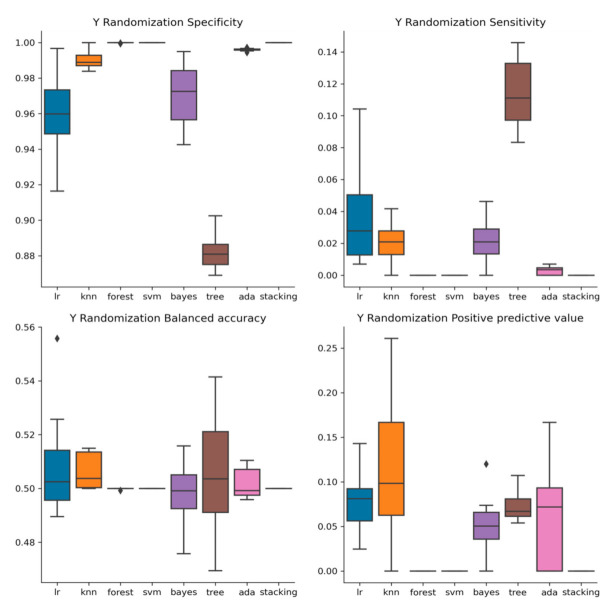
The performance of the QSAR models built with y-randomized data is inferior to that of the QSAR models developed with the non-permuted data set.

The models’ performance during y randomization tests was considerably worse than before randomly shuffling the response variable. Balanced accuracy was close to 50% in all cases compared to the original models, most of which had accuracy close to 60% and higher. These findings indicate that the models’ results are not obtained by chance and that there is an underlying relationship between the structure of the compounds and the response variable that the models have identified. Sensitivity dropped to 2% or lower and PPV to less than 10%, a majority of the substances being classified as inactive (Figure 3).

### 2.4. External Validation

We performed the external validation on the 1130 compounds from the ChEMBL database (left aside from the beginning for this purpose). The meta-model had a PPV of 40.00% on the external validation data set and balanced accuracy of 76.68% (i.e., 120 compounds were indeed active). A few individual models with higher PPV in nested cross-validation had higher PPV on the external validation set, but their sensitivity was considerably inferior to that of the meta-model (Table S4).

### 2.5. Outliers and Applicability Domain

Elimination of outliers from the dataset led to better performance of the meta-model: an increase by 2–4% at most in the different performance metrics used (BA, sensitivity, specificity, PPV).

By applying the method of F. Sahigara et al., 2013 [25], some 18–37 (1.59–3.27%) of the compounds from the external validation data set were found to be out of the AD of the individual models and 76 (6.73%) were out of the AD of the meta-model.

### 2.6. Descriptors

Different selection methods used selected different sets of descriptors as important for outcome prediction. VSA_EState1 (VSA EState Descriptor 1 (-inf < x < 4.78)) and BCUTi-1h (first highest eigenvalue of Burden matrix weighted by ionization potential) were the only ones that were selected by all four feature selection algorithms. The importance attributed to these two descriptors was relatively low, though (the highest rank for BCUTi-1h was 5, whereas for VSA_EState1 the highest rank was 20). Descriptors selected by three of the four selection algorithms were EState_VSA2 and ATSC2d (centered Moreau–Broto autocorrelation of lag 2 weighted by sigma electrons), only the latter being first in rank for the mutual information algorithm. The largest importance was attributed by the four selection algorithms to VR3_A (VR3 of adjacency matrix), ATSC2d, ATSC1dv (centered Moreau–Broto autocorrelation of lag 1 weighted by valence electrons) and AATS0dv (averaged Moreau–Broto autocorrelation of lag 0 weighted by valence electrons), respectively. Other features with high importance identified by the selection algorithms belonged to the classes of constitutional descriptors (number of heteroatoms, number of N atoms and number of halogen atoms) and the families of first highest eigenvalue of Burden matrix, centered Moreau–Broto autocorrelation and averaged and centered Moreau–Broto autocorrelation descriptors (Table S5).

In addition, the descriptors identified by the feature selection methods, we looked into the best-performing algorithms to get an insight about the most important chemical features associated with activity against *Pseudomonas aeruginosa*. The best performing KNN model on the native data set indicated (through the use of LIME) that higher values of ATSC2d, AATS7i and lower values of EState_VSA1 tend to associated with inactivity, whereas active compounds have lower values of AATS7i and higher values of EState_VSA1 and AATS4i (Figure S1). Αnother KNN model with similar performance showed that inactive compounds tended to have lower SMR_VSA3 and higher Μ-DEC33 and ATSC2d; instead, active compounds tended to have high SMR_VSA3, BCUTi-1h and BCUTs-1h (Figure S2). A third KNN model indicated that compounds with higher VSA_EState9 and lower SMR_VSA3 are inactive, whereas those with low SaasC (two aromatic and one single carbon) and ATSC3v and high BCUTi-1h and SMR_VSA3 were active (Figure S3). For each of the features, though, the impact was small (about 2–3% influence for each feature) in the KNN and decision trees.

The best performing logistic regression (through the use of Eli5) indicated that higher values of ATSC7d and BCUTi-1h were associated with higher activity, whereas NaaaC (N meta to C) and ATSC2d were associated with lack of activity (Figure S4).

Based on the ELI5, three decision-tree models with the native Saagar features indicated that the most relevant substructure associated with activity was the pattern atom-ring-atom-ring-atom-ring-atom (four generic atoms separated by three generic rings). However, the weight assigned to this substructure was relatively low (0.11–0.13). Other features identified more frequently by the decision-tree models seemed either not very specific (a nitrogen atom, or an aliphatic carbon with one hydrogen and two additional indifferent bonds, or an aromatic carbon with no hydrogen bond) or too specific (e.g., one nitrogen or oxygen without additional hydrogen + a chain of three carbon atoms with two carbonyl groups + one aliphatic carbon + three chlor/fluor atoms etc.) (Figures S5–S7). No aromatic carbon bonded on two sides with S, O or N, or more than six non-aromatic atoms of O, N (valence 3), P (valence 3), F or Cl, tend to be active (4–5% effect on prediction) (Figure S5). The effect for each of these features was around 9–10% in the random forest model built with the downsampling data set and varied between 5–9% for each in the case of knn models built with the SMOTE data set. The SMOTE best performing knn models also identified the absence of a methylene bonded with another aliphatic carbon and with one of (O, N, P, S, F, Cl, Br, I) as associated with activity (7–14%).

## 3. Discussion

*Pseudomonas aeruginosa* is one of the six ESKAPE microbial species that are justifiably worrying for the public health landscape today [4] and a worldwide need to develop new antibiotics active against this bacterial species is increasingly pressing [7]. In the available literature, QSAR models have been reported for substances potentially active against *P. aeruginosa:* local 2D-QSAR models for specific chemical classes (indolylpyrimidines [26], N-octaneamino-4-aminoquinolines [27]), local 2-D [28] and 3D-QSAR models of compounds active against specific protein targets from *P. aeruginosa*, such as the UDP-3-O-(R-3-hydroxymyristoyl)-N-acetylglucosamine deacetylase (LpxC) [29]. A multi-tasking QSAR model (oriented to both predicting anti-*Pseudomonas* activities and ADMET properties of chemical compounds) has also been developed [30]. We have developed a set of global models using individual machine learning algorithms and an ensemble model to predict antimicrobial activity of chemical compounds against *P. aeruginosa* based on the inhibition zone values measured in disk diffusion tests. As stated in the introduction, such data are relatively abundant in public databases such as ChEMBL and they contain relevant information that is useful to be drawn to light through modeling such as QSAR. Probably because of the qualitative character of the measurements in this type of testing, such ChEMBL data sets have not been very appealing to the QSAR community up to date. This reluctance may mirror the hesitation of some laboratories to use the disk diffusion method, partly because of reported issues associated with disk quality or the inability of this method to provide an MIC value that can guide clinicians in their therapeutic approach [31], but many settings in the world continue to use such data [32]. Our results have shown that models with reasonable performance can be built and employed for virtual screening purposes, although their performance may be lower than that of those built with MIC, MIC90 or IC50 (which may be less noisy). Of the latter, however, MIC90 and IC50 tend to be less represented (smaller data sets), resulting in a different category of shortcomings.

One issue in using IZ measurements as an outcome variable is finding an appropriate cut-off level for the classification of compounds in active and inactive. We used a cut-off based on the literature data and clinical breakpoints established by competent organizations (CLSI, EUCAST), indicating that for an important number of currently, used antibiotics the threshold between resistance and sensitivity or in some instances “areas of technical uncertainty”, is 25 mm or lower [33,34,35]. However, this cut-off is not without problems, since clinical breakpoints used in the medical practice are substance-specific, but they are only available for a small number of authorized antibiotics, but not for the largest number of compounds tested for scientific purposes outside a clinical setting. Moreover, antimicrobial susceptibility testing breakpoints are established taking into account not only in vitro results of a large number of microbial isolates, but also pharmacokinetic, pharmacodynamics and clinical considerations specific for each antibiotic [36]. Furthermore, experts in the field of disk diffusion testing emphasize that IZ is dependent on the diffusion rate (through the agar gel) of the tested compound, which in its turn is dependent on certain drug features, such as its size or partition coefficient [24]. These factors make that any uniform cut-off threshold (one not individualized for each compound) should result to some extent in misclassification and this might explain why the performance of our models, while decent (mean balanced accuracy in nested cross-validation 72.61%), was not particularly impressive.

Given the limitations of a binary cut-off, would not regression modeling be preferable? In theory, the answer would be yes, but as already discussed, the results provided by the disk diffusion method are only qualitative and usually, “the results have large variations” [37]. Our exploration of regression machine learning models with several algorithms indicated RMSE values around 6 mm in nested cross-validation (with hyperparameter tuning in the inner loop), too large in our view to be of practical relevance.

In terms of balanced accuracy, among individual models, the best performance was provided by KNN, logistic regression and decision tree classifier, but the ensemble method had superior results to individual models in this respect (the only model with BA generally over 70% in nested cross-validation, as well as on the external test set). KNN models have been successfully applied for other QSAR models, e.g., for different histone deacetylase inhibitors [38,39] or to predict binding affinity for different G-Protein Coupled Receptors (GPCRs) [40]. Logistic regression with regularization, although a relatively simple algorithm, has been shown to have similarly good performance as more sophisticated algorithms in QSAR models [41]. On our dataset the decision trees classifier, somewhat surprisingly, had better results than random forests, although in other cases, the latter has shown superior performance [42]. SVM and AdaBoost classifiers, which for other datasets have been very successful [43,44,45,46], in our case, did not perform as well. The balancing techniques (downsampling, naïve random oversampling and SMOTE) generally contributed little to the improvement of performance, particularly in models built with the different fingerprints. In the case of molecular descriptors, a small number of models had slightly better performance on the external validation set (because of the artificial nature of these data sets, the nested cross-validation data have little relevance in assessing the performance of such models).

The key descriptors used in building the models, selected with the help of four different selection algorithms, belonged to the families of adjacency matrix, constitutional descriptors, first highest eigenvalue of Burden matrix, centered Moreau–Broto autocorrelation and averaged and centered Moreau–Broto autocorrelation descriptors. Adjacency matrix descriptors have also been previously used [47,48] to predict the antimicrobial effects of different chemical compounds. To a limited extent the Moreau–Broto autocorrelation descriptors [49], but we could not find in the literature references to the first highest eigenvalue of the Burden matrix in relationship with the antimicrobial activity. The other descriptors identified by analyzing the better performing models, including the Saagar substructures, were not previously reported in the literature as associated with antimicrobial activity.

## 4. Materials and Methods

**Figure 4:**
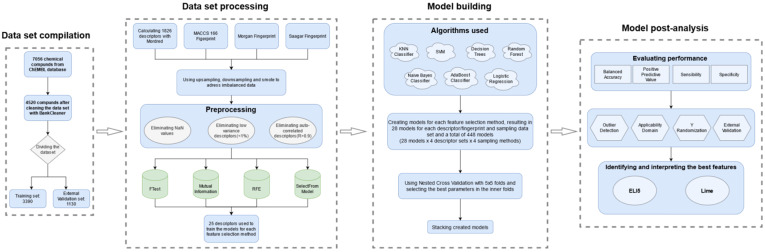
Graphical workflow of the modeling process used in this study.

A graphical workflow of the modeling process is shown in Figure 4.

### 4.1. The Dataset

The data set used for model building was obtained from the ChEMBL database by searching “Pseudomonas aeruginosa” in the targets section and downloading all compounds associated with an IZ value. The initial data set consisted of 7056 compounds SMILES chemical structures (provided by ChEMBL), which were converted into 2D structures (sdf) using Bank Formatter 2017. They were filtered using the Bank Cleaner 2017 in order to remove inorganic compounds, mixtures, empty structures, salts and duplicates; both services were provided by the FAF-Drugs4 program [50,51] and resulted in a dataset of 4520 chemical compounds. The latter were classified into 2 groups, using a cut-off of 25 mm: compounds with an IZ of more than 25 mm were labeled as 1 (active) and compounds with IZ under 25mm as 0 (inactive). This dataset was divided in two subsets: one used for model development and performance assessment through nested cross-validation (3390 compounds—Table S1) and the other used for external validation purposes (1130 compounds—Table S2). Structural outliers were identified and removed from the training data set, as recommended in the QSAR literature [52] and as described below. Among the 3390 compounds (of which 261 were active and 3226 inactive) of the training set, 164 outliers were identified with the isolation forest algorithm and eliminated, leaving a final training data set of 3226 chemical compounds, of which 2986 were inactive and 240 were active. For the dissimilarity plot, MACCS fingerprints were computed with the R “RCpi” package [53], Tanimoto coefficients with the help of the “IntClust” R package [54], whereas the dissimilarity plot was generated with the “seriation” R package [55].

### 4.2. Descriptors and Feature Selection

Using Mordred [56], a Python package, we computed a total of 1826 descriptors. MACCS keys and Morgan/Circular fingerprints (using a radius of 2 and 1024 bits) were also computed in Python, using the RDKit library [57]. The same modeling approach was applied for descriptors and molecular fingerprints. Because very recently the *Saagar* substructures have been described as “a better choice for building interpretable predictive in silico models and read-across protocols”, for their interpretable and efficient character [58], we also used these features. We obtained the Saagar features as a text file in a SMARTS format, under an academic license and used the “rcdk” R package [59] to compute the fingerprints based on these features.

The initial dataset of descriptors (and, correspondingly, fingerprints) was filtered by eliminating those with invalid values, highly correlated (R > 0.9) and those with low variance (<1%). We were thus left with a total of 199 descriptors.

Further feature selection was performed on these in order to reduce noise and eliminate redundancy by lowering the dimensions of the data [60]. We used four feature selection methods: two univariate methods (f-test and mutual info classifier), along with recursive feature elimination using a cross-validation loop to select the best number of features and, finally, feature selection using “SelectFfromModel” with decision trees as the estimator. 

For univariate feature selection, the “select k best method” was used, which eliminates all features except those with the highest score computed with a score function, e.g., a function using ANOVA f-value. The f-test assesses the degree of association between two variables by computing the ratio of the two variances, between classes and within classes, as in the classical ANOVA test [61,62]. Mutual information is a tool used to assess the degree of statistical independence among variables, with two fundamental properties: (a) ability to capture not only linear but also non-linear relationships and (b) is invariant under any invertible transformation of the variables used as features in the modeling process [63]. The mutual information classifier used by scikit-learn for feature selection is implemented based on entropy estimation [64,65]. Recursive feature elimination is, in theory, superior to the previously mentioned two filter methods. It is based on an iterative procedure implying training of a classifier, ranking all the features using a specific criterion and removing the feature with the lowest rank [66]. “SelectFfromModel” is a versatile meta-transformer implemented by scikit-learn, that removes features based on a threshold that can be predefined or found with the help of built-in heuristics [60]. 

By using multiple iterations, we determined that the optimal number of features for building our models was 25. Performance improved as we selected more features until this point, after which it began to flatten or even decrease, regardless of the increase in the number of features selected.

### 4.3. Classification Algorithms

The following algorithms were used to build classification models: support vector classifier, K nearest neighbors, random forest classifier, decision tree classifier, AdaBoost classifier, logistic regression and naïve Bayes classifier. All algorithms were implemented in Python (version), using the scikit-learn package.

K nearest neighbors classifier is based on the notion that a target can be assigned its neighbors’ label, using a similarity measure such as the Euclidian distance, Hamming distance etc. [67]. The shortest the distance between a number k of data points to the target, the more similar those points are to the target and a mere majority vote decides the label. The k parameter has a strong sway over the model decision and its tuning is needed to achieve a balance between over- and under-fitting [68,69]; the values we used ranged between five and eight.

Decision tree is a classification method that labels a data set based on a tree of dichotomic rules [70]. In the learning phase, the rules are derived (tree generation) and in an accuracy verification phase, random data taken from the training set is tested and rules are adjusted in order to decrease the tree size (tree pruning); in the end the unlabeled data points are classified with the rules thus developed and tested [70,71]. Simplicity, transparency, easiness to understand and to implement [72,73] are key advantages of the decision tree classifier. The key parameter influencing the tree’s performance is its maximum depth, as it decides its complexity [74]; in our models, this parameter had values between two and four.

Random forest is an ensemble method that builds multiple decision trees to assign a new data point to a class by a simple majority vote [75]. From statistical and computational perspectives, random forests have multiple strengths, including powerful discriminative abilities, that make them appealing for many applications [76]. The correlation between individual trees is low due to random feature selection of each tree, which results in superior efficiency of the classifier [77]. This classifier’s key parameters are the number of trees and their depth (controlled via minimum node size) [78]; our models used between 100 and 300 trees with depths between 10 and 50.

The support vector machine (SVM) is an algorithm widely used for classification purposes, based on identifying the optimal hyperplane to separate observations into the labeled classes [79]. This hyperplane is found with the help of the closest data vectors of the two classes (in the case of binary classification), which are known as “support vectors” [80]. We used the radial basis function kernel and tuned the C and gamma values between 0.5–10 and 0.1–1, respectively.

AdaBoost (short for adaptive boosting) is an ensemble method that integrates multiple weak classifiers (models) to build a strong one [81]. Multiple models are constructed sequentially, starting with equal weights for each observation from the training set and then gradually adjusting the weights; after multiple iterations, the results [82]. The key hyperparameters are the number of estimators (we used values between 100 and 400) and the learning rate (we used 0.2–0.5).

Gaussian Naive Bayes (GNB) classifier is much faster than other widely used algorithms (such as SVM or even logistic regression) because it hypothesizes a diagonal covariance matrix between variables, thus avoiding the computation of the full covariance matrix [83]. The algorithm assumes Gaussian distribution of the classes, computing z-scores and converting them to *p* values using the Bayes’ theorem, i.e., computing the probability of an observation belonging to class A or class B, given the observed data [84]. The algorithm has a “naïve” approach, not modeling the covariance between features and assuming Gaussian distribution and the assumptions on which it is based are not necessarily valid. It may work relatively well in a surprising number of cases (but not in all and not as powerful as more sophisticated algorithms) [84].

Logistic regression is a statistical method often employed in the machine learning applications for binary classification, having simplicity and excellent interpretability as its key advantages [85]. It computes the probability p = 1/(1 + e^−t^), where t = b0 + b_1_x_1_ + b_2_x_2_ + … + b_n_x_n_ [86]_._ To make predictions, a decision threshold is used (the default value being 0.5, but any other threshold may be used to optimize performance metrics) [87].

In the field of machine learning (unlike statistics), logistic regression is used with a regularization function, meant to avoid overfitting; a cost parameter C is employed for this purpose, for which we used values ranging from 0.1 to 100. We used l2 regularization and ‘lbfgs’ solver; we also used the ‘balanced’ mode for the ‘class_weight’ parameter, this means that the algorithm will automatically adjust class weights inversely proportional to class frequencies.

Hyperparameter tuning was conducted in the inner loop of nested cross-validation by selecting parameters from a specified parameter grid.

A total of 28 models (from 32 built) were stacked to build a meta-model [88], using eight algorithms and four feature selection methods. The algorithm used for stacking was logistic regression. It used the predicted probabilities from the individual models to make the final prediction. The stacked model used nested cross-validation as well; for stacking, we used the stacking classifier provided by sci-kit learn.

Because the data set is imbalanced (with a ratio of active: inactive compounds of 12.44:1), we tuned hyperparameters using balanced accuracy as the primary performance metric and whenever the algorithm permitted, we used options to take into account the class imbalance (i.e., using class weights or an appropriate loss function). In addition, we applied the techniques of naïve random oversampling (using the “RandomOverSampler” function), downsampling (with the “ClusterCentroids” function) and SMOTE (*synthetic minority oversampling technique* with the “BorderlineSMOTE” function), with the help of the “imbalanced-learn” Python library, v. 0.8.0. [89]

Machine learning models tend to be “black boxes” and have for a long time been criticized for their lack of interpretability [90]. However, the last years have witnessed the occurrence of several methods and software implementation intended to help with the interpretation of machine learning models. To get access to the interpretability of the models developed, we made use of two python libraries: ELI5, which returns information on the weight attributed by the model to each of the features used [91] and ”lime” [92], which implements the so-called method of local interpretable model-agnostic explanations.

### 4.4. Performance Evaluation

The performance was assessed using nested cross-validation with five folds in the inner loop and five folds in the outer loop. Nested (double) cross-validation outperforms simple (k-fold) cross-validation and hold-one-out validation in terms of both avoiding overfitting and underfitting [93,94].

We computed the following metrics: balanced accuracy (BA), sensitivity, specificity and positive predictive value (PPV). Our interest was to have higher certainty about the activity of the identified substances (i.e., high specificity) while at the same time preferring not to lose too many potentially active substances (i.e., reasonable sensitivity). All metrics were computed with the classification report method offered by sci-kit learn [60]. Different seed numbers were used to assess the models’ performance in the nested cross-validation setting (5 times).

A y randomization test [95] was performed to verify to what extent the results obtained are likely to have been obtained by mere chance. This test was done by randomly scrambling the activity label and rebuilding all the models using the same methods as before; this process was applied a hundred times and the same metrics were used for performance evaluation. The results of the y randomization test should be considerably worse than the results of the models using the unshuffled data.

### 4.5. Outlier Detection and Applicability Domain

An outlier is defined as a data point that differs significantly from or appears inconsistent with the rest of the data points [96]. The presence of outliers in a data set can be a problem when building a model (as it may unduly influence model parameters resulting in wrongly specified models), but outliers may also contain important information. Therefore, a decision to remove them should be well-founded and not for the mere purpose of having models with an apparent better performance [96]. Detection of outliers was carried out using the isolation forest algorithm as implemented in scikit-learn [60] because in a complex benchmarking assessment, this algorithm had the best performance [97]. 

The applicability domain (AD) is the vector space where a mathematical model (such as a QSAR one) can be applied with reasonable confidence, in other words, the interpolation region [98]. For a new (test) compound to be inside the applicability domain, it has to be sufficiently similar to the compounds from the training set used to develop the model [99]. To determine the AD, the method proposed by F. Sahigara et al., 2013 [25] was used.

### 4.6. External Validation

The external validation dataset (1130 compounds) consisted of 1043 inactive and 87 active compounds. The individual models and the meta-model were tested on this data set after determining which compounds were inside the models’ AD. The same metrics were used to assess their performance.

## 5. Conclusions

Models with reasonably good performance can be built and employed for virtual screening purposes using disk diffusion results (inhibition zone), but they tend to be noisier (less precise) and consequently their performance may be slightly lower than that of those built with other measurements, such as MIC, MIC90 or IC50 (which may be less noisy), but they could be integrated with data based on such endpoints.

## Data Availability

Data supporting the results in this paper have been provided as supplementary information files. More information can be obtained from the correspondence author.

## Supplementary Materials

Table S1: Training data set for molecular descriptors, Table S2: External validation data set for molecular descriptors, Table S3: Mean results in nested cross-validation (5 runs) for models built with molecular descriptors, Table S4: External validation results for models built with molecular descriptors, Table S5: Descriptors used for model building by feature selection method, Table S6: Nested_cross_validation results for models built with the downsampling technique, Table S7: Nested_cross_validation results for models built with the naïve randome oversampling technique, Table S8: Nested_cross_validation results for models built with SMOTE, Table S9: External validation results for models built with the downsampling technique, Table S10: External validation results for models built with the naïve random oversampling technique, Table S11: External validation results for models built with SMOTE, Table S12: Nested_cross_validation results for models built with MACCS fingerprints, Table S13: External validation results for models built with MACCS fingerprints, Table S14: Nested_cross_validation results for models built with the downsampling technique (MACCS fingerprints), Table S15: External validation results for models built with the downsampling technique (MACCS fingerprints), Table S16: Nested_cross_validation results for models built with the naïve random oversampling technique (MACCS fingerprints), Table S17: External validation results for models built with the naïve random oversampling technique (MACCS fingerprints), Table S18: Nested_cross_validation results for models built with SMOTE (MACCS fingerprints), Table S19: External validation results for models built with SMOTE (MACCS fingerprints), Table S20: Nested_cross_validation results for models built with Morgan fingerprints, Table S21: External validation results for models built with Morgan fingerprints, Table S22: Nested_cross_validation results for models built with the downsampling technique (Morgan fingerprints), Table S23: External validation results for models built with the downsampling technique (Morgan fingerprints), Table S24: Nested_cross_validation results for models built with the naïve random oversampling technique (Morgan fingerprints), Table S25: External validation results for models built with the naïve random oversampling technique (Morgan fingerprints), Table S26: Nested_cross_validation results for models built with SMOTE (Morgan fingerprints), Table S27: External validation results for models built with SMOTE (Morgan fingerprints), Table S28: Nested_cross_validation results for models built with Saagar fingerprints, Table S29: External validation results for models built with Saagar fingerprints, Table S30: Nested_cross_validation results for models built with the downsampling technique (Saagar fingerprints), Table S31: External validation results for models built with the downsampling technique (Saagar fingerprints), Table S32: Nested_cross_validation results for models built with the naïve random oversampling technique (Saagar fingerprints), Table S33: External validation results for models built with the naïve random oversampling technique (Saagar fingerprints), Table S34: Nested_cross_validation results for models built with SMOTE (Saagar fingerprints), Table S35: External validation results for models built with SMOTE (Saagar fingerprints), Table S36: Training set for MACCS fingerprints, Table S37: External validation set for MACCS fingerprints, Table S38: Training set for Morgan fingerprints, Table S39: External validation set for Morgan fingerprints, Table S40: Training set for Saagar fingerprints, Table S41: External validation set for Saagar fingerprints, Figure S1: LIME_interpretation_of KNN model built with molecular descriptors and_FTest selection method, Figure S2: LIME_interpretation of_KNN model built with molecular descriptors and Mutual_information selection method, Figure S3: LIME_interpretation of_KNN model built with molecular descriptors and_SelectFromModel selection method, Figure S4: ELI5 interpretation_of logistic_regression_models built witmolecular descriptors; Figure S5: ELI5 interpretation of decision tree model built with Saagar fingerprints and SelectFromModel selection method; Figure S6: ELI5 interpretation of decision tree model built with Saagar fingerprints and Mutual Information selection method; Figure S7: ELI5 interpretation of decision tree model built with Saagar fingerprints and Recursive Feature Eleimination selection method.
