# Supplementary figures and images for: QSAR Models for Active Substances against Pseudomonas aeruginosa Using Disk-Diffusion Test Data
Source: Molecules. 2021 Mar 19;26(6):1734. doi: 10.3390/molecules26061734 (PMC8003670; doi:10.3390/molecules26061734)

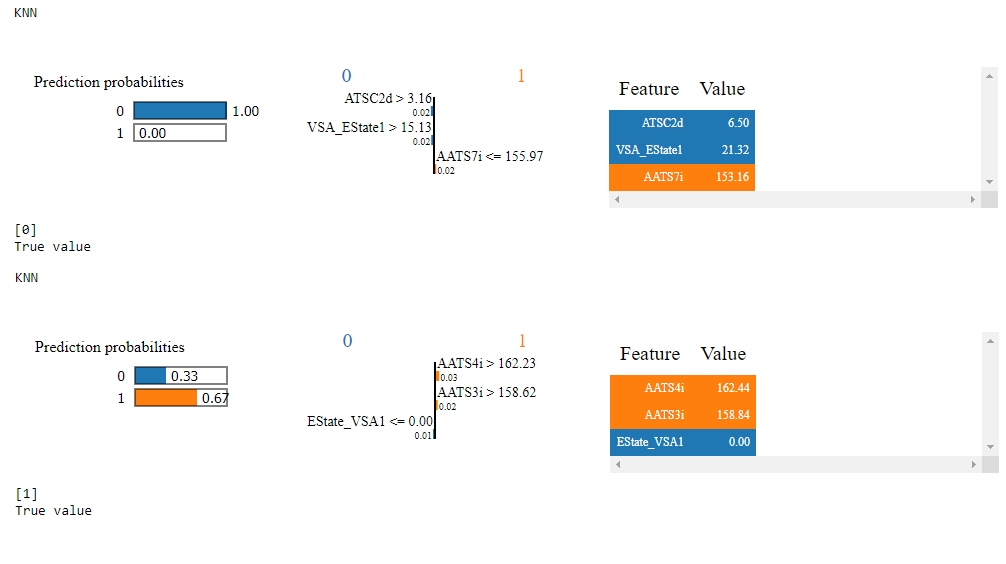

Supplement: Supplementary file 1 [file molecules-26-01734-s001.zip › Supplementary materials/Fig. S1. LIME_interpretation_KNN_FTest.png]

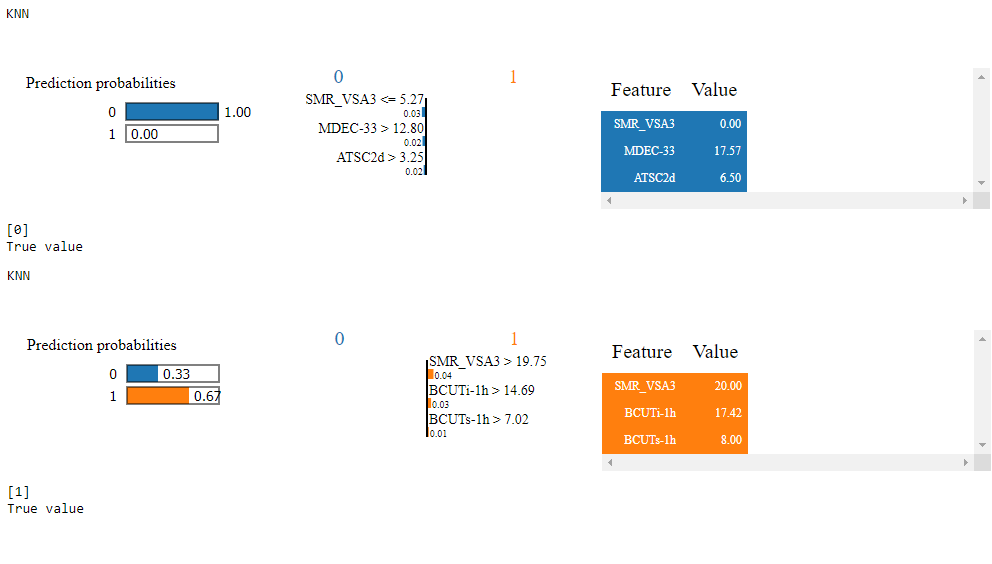

Supplement: Supplementary file 1 [file molecules-26-01734-s001.zip › Supplementary materials/Fig. S2. LIME_interpretation_KNN_Mutual_information.png]

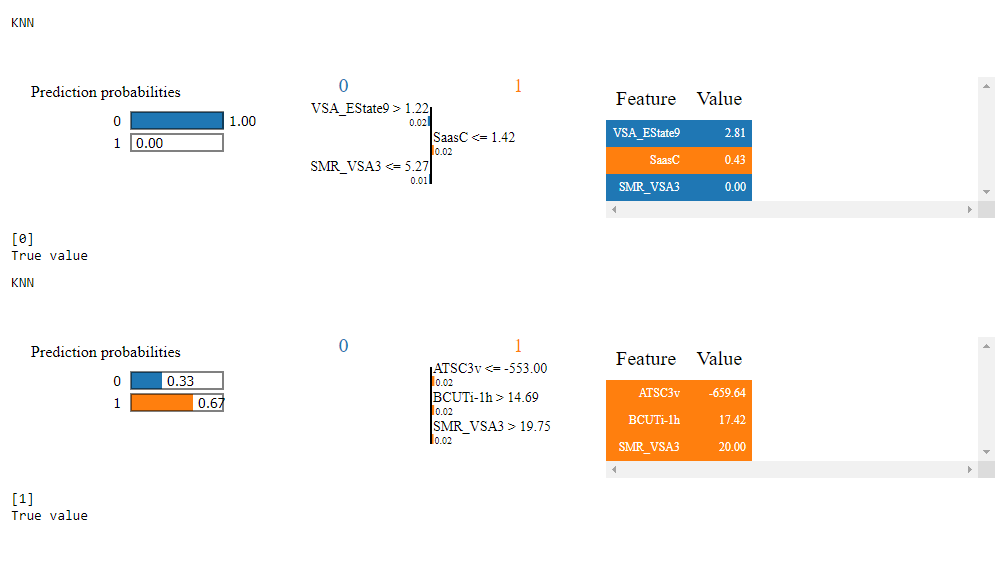

Supplement: Supplementary file 1 [file molecules-26-01734-s001.zip › Supplementary materials/Fig. S3. LIME_interpretation_KNN_SelectFromModel.png]

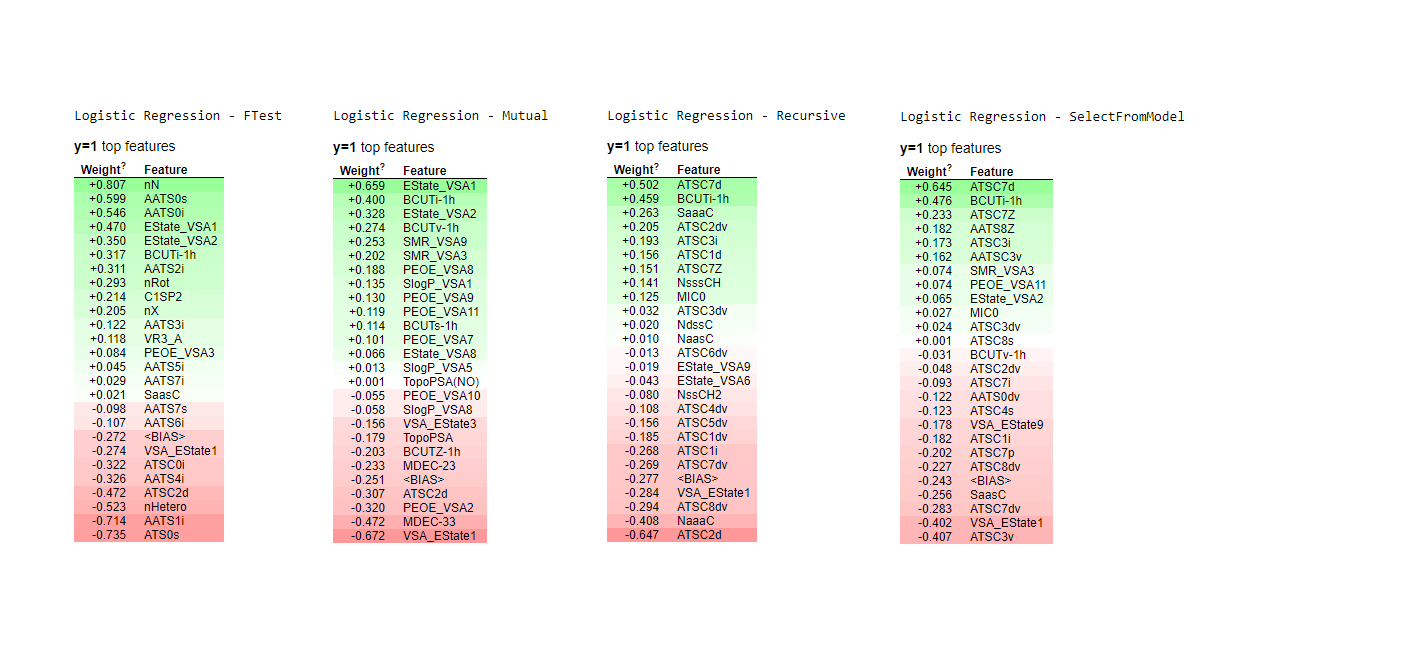

Supplement: Supplementary file 1 [file molecules-26-01734-s001.zip › Supplementary materials/Fig. S4. ELI5_interpretation_logistic_regression_models.png]

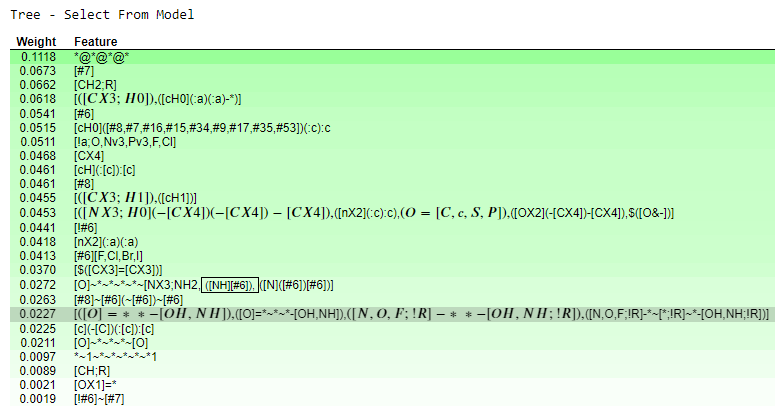

Supplement: Supplementary file 1 [file molecules-26-01734-s001.zip › Supplementary materials/Fig. S5. ELI5_interpretation_Decision_Tree_SelectFromModel.png]

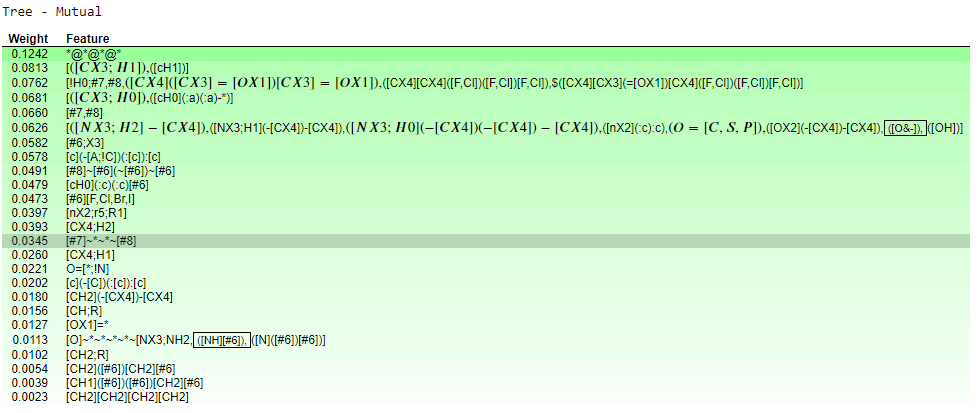

Supplement: Supplementary file 1 [file molecules-26-01734-s001.zip › Supplementary materials/Fig. S6. ELI5_interpretation_Decision_Tree_Mutual_Information.png]

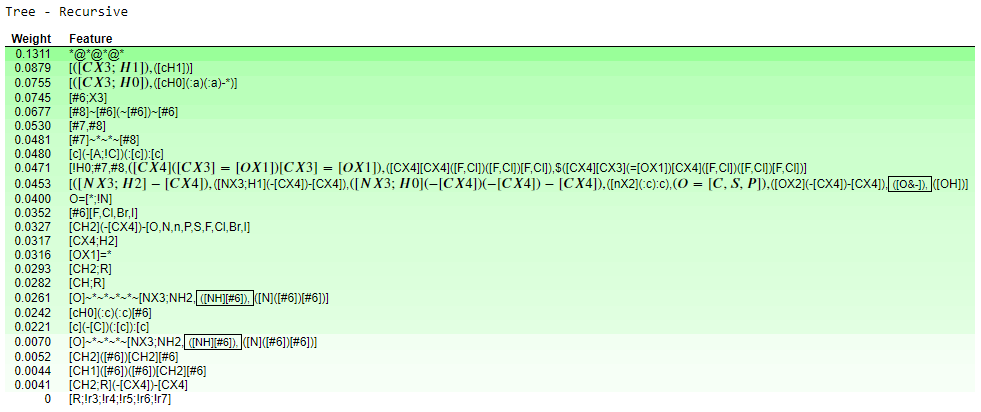

Supplement: Supplementary file 1 [file molecules-26-01734-s001.zip › Supplementary materials/Fig. S7. ELI5_interpretation_Decision_Tree_Recursive_FE.png]
